# A85 PERCEIVED FOOD INTOLERANCES IN PATIENTS WITH INFLAMMATORY BOWEL DISEASE COMPARED TO HEALTHY CONTROLS

**DOI:** 10.1093/jcag/gwad061.085

**Published:** 2024-02-14

**Authors:** R Dang, D Boron, J Linton, P Mundra, N Narula, A Caminero

**Affiliations:** McMaster University Faculty of Health Sciences, Hamilton, ON, Canada; McMaster University Faculty of Health Sciences, Hamilton, ON, Canada; University of Toronto, Toronto, ON, Canada; McMaster University Faculty of Health Sciences, Hamilton, ON, Canada; McMaster University Faculty of Health Sciences, Hamilton, ON, Canada; McMaster University Faculty of Health Sciences, Hamilton, ON, Canada

## Abstract

**Background:**

Inflammatory bowel disease (IBD) patients commonly exhibit food intolerance. However, the precise dietary triggers and mechanistic pathways responsible for food intolerances are unknown. The characterization of the main offending foods in intestinal inflammation could guide targeted dietary interventions in IBD management.

**Aims:**

To examine self-reported food intolerances in IBD participants compared to healthy controls.

**Methods:**

IBD participants (18-75 years old with a confirmed ulcerative colitis (UC) or Crohn’s disease (CD) diagnosis) were recruited from the Gastroenterology clinic at McMaster University from August 2021 to September 2023. Healthy controls (18-75 years old with medical condition) were recruited through recruitment posters at McMaster University. Participants completed questionnaires pertaining to self-reported food intolerances, gastrointestinal rating symptoms, demographic information, and past medical conditions.

**Results:**

A total of 80 IBD participants (48 CD and 32 UC) and 26 controls completed the questionnaires. We found that IBD participants (88% CD and 90% UC) reported at least one food intolerance compared to controls (30%). The mean number of food intolerances IBD participants reported was 3 (SD=1.78). The most common adverse reactions were reported to dairy (60% CD and 63% UC), wheat (37% CD and 40% UC), peanuts/tree nuts (27% CD and 25% UC), caffeine (27% CD and 25% UC) and fiber (22% CD and 28% UC). The rates of intolerances to all of the food groups were higher than reported by controls. The most common symptoms include discomfort (75% CD and 50% UC), bloating (66% CD and 62% UC), diarrhea (62% CD and 56% UC) and pain (56% CD and 37% UC) when consuming offending foods. About half of IBD participants had these symptoms before their diagnosis and 30% reported worsening of intolerances after their IBD diagnosis.

**Conclusions:**

Our current findings reveal that IBD participants commonly report food intolerances that are associated with symptomatology. Common offending foods include dairy, wheat, peanuts/tree nuts and caffeine. IBD participants commonly experience discomfort, bloating and diarrhea when eating offending foods, which can persist after diagnosis. Ongoing studies will provide insights into the relationship between stool bacterial composition and food intolerance symptoms.

**Funding Agencies:**

CCC

